# 3D Printing to Support the Shortage in Personal Protective Equipment Caused by COVID-19 Pandemic

**DOI:** 10.3390/ma13153339

**Published:** 2020-07-27

**Authors:** Mostapha Tarfaoui, Mourad Nachtane, Ibrahim Goda, Yumna Qureshi, Hamza Benyahia

**Affiliations:** 1 ENSTA Bretagne, IRDL—UMR CNRS 6027, F-29200 Brest, France; yumna.qureshi@ensta-bretagne.org (Y.Q.); h.benyahia.dms@gmail.com (H.B.); 2 Arts et Metiers Institute of Technology, University of Bordeaux, CNRS, Bordeaux INP, INRAE, I2M Bordeaux, F-33400 Talence, France; mourad.nachtane@u-bordeaux.fr (M.N.); ibrahim.goda@u-bordeaux.fr (I.G.)

**Keywords:** COVID-19, novel coronavirus, medical devices, personal protective equipment, additive manufacturing/3D printing, technical considerations, material biocompatibility

## Abstract

Currently, the emergence of a novel human coronavirus disease, named COVID-19, has become a great global public health concern causing severe respiratory tract infections in humans. Yet, there is no specific vaccine or treatment for this COVID-19 where anti-disease measures rely on preventing or slowing the transmission of infection from one person to another. In particularly, there is a growing effort to prevent or reduce transmission to frontline healthcare professionals. However, it is becoming an increasingly international concern respecting the shortage in the supply chain of critical single-use personal protective equipment (PPE). To that scope, we aim in the present work to provide a comprehensive overview of the latest 3D printing efforts against COVID-19, including professional additive manufacturing (AM) providers, makers and designers in the 3D printing community. Through this review paper, the response to several questions and inquiries regarding the following issues are addressed: technical factors connected with AM processes; recommendations for testing and characterizing medical devices that additively manufactured; AM materials that can be used for medical devices; biological concerns of final 3D printed medical parts, comprising biocompatibility, cleaning and sterility; and limitations of AM technology.

## 1. Introduction and Scope

The novel coronavirus disease (SARS-CoV-2) has caused an acute reduction in world supplies of personal protective equipment (PPE) due to the increased demand and significant disruptions to the global supply chain, leaving many staff and patients without protection. Epidemic diseases that spread across a large region of the world can pose a challenge to healthcare resources around the world. Basic measures such as personal hand cleaning and social distancing are crucial to reduce the spread of infections. However, according to the World Health Organization (WHO) guidelines, the personal protective material is indispensable for all healthcare workers. Currently, epidemic crisis situations can lead to a lack of protective face masks for health care providers such as doctors and nurses in hospitals and other health care settings. As coronavirus infection continues to spread rapidly, hospitals around the world are in urgent need of the necessary medical equipment to treat patients. Mechanical ventilators are also one of these essential items needed during this pandemic which has a severe shortage of supply. This pandemic situation will demonstrate emerging requirements for greater flexibility in approach, such as for in situ 3D printing technique of medical devices in healthcare. For medical devices including PPE, 3D printing is characterized by the advantage of easing the fabrication of any geometric or complex shape structures that will not be readily practicable using conventional non-additive manufacturing (AM) methods. Considering that 3D printing is a potential saviour to dwindling medical supplies in the fight against coronavirus, the present contribution will address and discuss several aspects related to this technology that could play an important role in the domain of medical products and devices. An up-to-date review will initially be conducted to provide an overview and categorization of 3D printed medical products and devices. The main focus is to determine the capacity of AM to provide exceptional benefits to humanity within the medical healthcare supplies sector. With the broadly accepted benefits belonging to the use of AM in healthcare applications, there are also some major limitations of additively manufactured parts, hence the advantages and limitations of this technology are subsequently explored. In terms of strengths and weaknesses, opportunities and threats (SWOT) analysis of 3D printing in the healthcare field is next performed. The ongoing research for different materials used in 3D printing towards medical applications is also highlighted and analyzed. As an important goal advocated in the present work, the implications of the COVID-19 pandemic on the global AM industry are explored. In this context, 3D printing companies as well as academia over the world that embarked helping in the production of medical devices, accessories, components, and/or parts using 3D printing technology to combat the shortage of PPE for healthcare professionals in their fight against COVID-19, are profoundly surveyed. A comprehensive overview of the materials mostly used to print the medical supplies such as face masks for medical providers battling COVID-19 is additionally provided. Moreover, throughout the rest of this contribution, the following technical issues are discussed: technical factors connected with 3D printing processes, recommendations to test and characterize the medical parts that additively fabricated, and biological issues related to final 3D printed medical products, comprising biocompatibility cleaning and sterilization.

## 2. Shortage in Medical Supplies Against COVID-19

**Figure 1:**
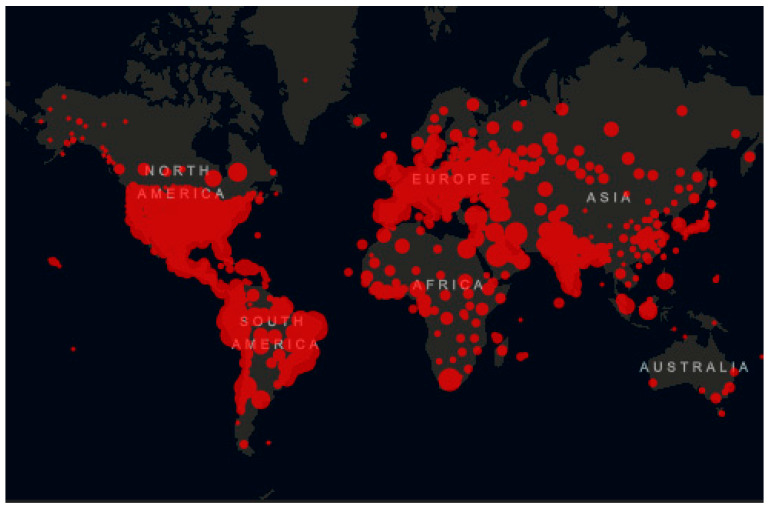
Distribution map of the virus worldwide as of 1 July.

In recent days, almost the entire world has been affected by a pandemic that has claimed many victims. Right now, the COVID-19 causing virus continues to spread. According to the Center for Systems Science and Engineering (CSSE) at Johns Hopkins University, as of midnight on 1 July 2020, 10,509,749 cases have been confirmed, including 512,071 deaths. The majority of deaths are in USA with 127,457 deaths followed. In Brazil, on the same date, 1,402,041 cases were confirmed, of which 59,594 people died [1]. Figure 1 shows the distribution map of the virus worldwide as of 1 July.

**Figure 2:**
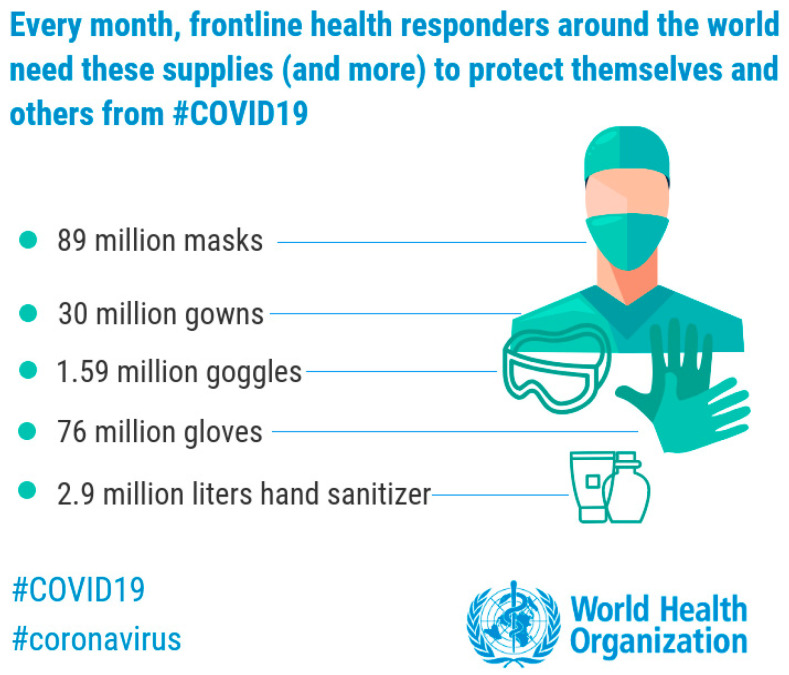
Shortage of medical devices against COVID-19 [2].

In this critical situation, the World Health Organization (WHO) warns of a global shortage of masks and other protective equipment against COVID-19. According to WHO prediction, an estimated 89 million medical masks are requested for the COVID-19 response each month. For examination gloves goes up to 76 million, while the international request for goggles stands at 1.59 million per month, Figure 2. The WHO published a statement calling on governments to expand incentives for industry to ramp up manufacturing, as well as ease restrictions on the export and distribution of PPE and other medical devices [2]. Currently, China manufactures about half of the world’s face masks and it has started shipping masks to other countries as part of goodwill packages.

**Figure 3:**
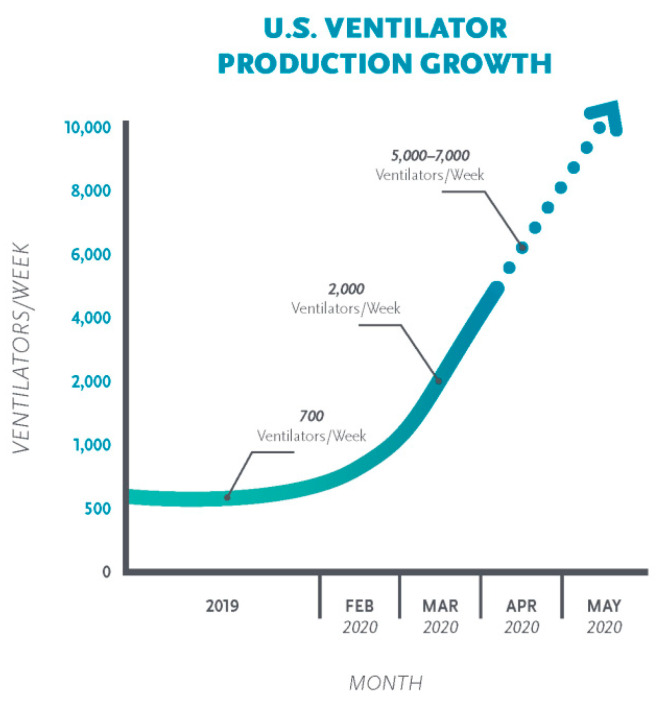
Production growth of ventilators in the USA [4].

On the other hand, there is a large variety of assessments of the quantity of ventilators one will necessity to care for patients with COVID-19, from various hundred thousand to as many as a hundred million. The estimation varies depending on the number, rate, and seriousness of contagion. In the United States, current estimates of the number of ventilators range from 60,000 to 160,000, depending on whether those that have only partial functionality are included [3]. Figure 3 presents the production growth of ventilators per week in the United States.

In this context, AM can make a difference and respond to the urgency of the spread of this epidemic. Several trades are involved to face the global health crisis linked to COVID-19. The players in AM are mobilizing their know-how, their capacity for innovation, their ideas and their energy to develop new means, prototypes of masks, protective visors, or even 3D printed valves to transform diving masks into emergency respirators. AM companies have responded to the call in droves, citing speed of production and distributed manufacturing networks as key factors.

## 3. AM Technology Against COVID-19

### 3.1. An Up-To-Date Review of the AM Technology in Medical Engineering

The development of civilization obliges researchers and engineers to find the new solutions and technologies requisite to optimize the manufacturing process of the product in all human activities especially in medical applications [5]. In this context, AM is a breakthrough technology that is changing the way products are made. One of the specificities of health care is that it is essential to know how to adjust to each patient. The tools or devices used in surgery or patient treatment often need to be customized. On top of that, these devices are frequently pricey and enough restricted. These limitations are compatible with the use of 3D printing, which makes it feasible to manufacture unique, personalized products at a rational price. It is for these reasons, among others, that 3D printing of medical parts has grown very rapidly within this industry. It’s very important to report that the AM revolution stems from several benefits over traditional manufacturing processes such as mass customization, complex designs and geometries, waste reduction, supply chain simplification, faster time-to-market, drastic assembly reduction, reduction of weight (topology optimization) and low volume manufacturing [6]. It is for these reasons in particular that 3D printing of medical parts has increased rapidly within this field.

The use of AM in medicine enables personalization of patient care. Health specialists display the patient’s injuries in 3D and are thus capable to simulate the preoperative surgical process to print artificial implants [7]. A promising field is in new medication delivery systems, in which it is likely to precisely control the quantity of dosed drug depending on individual patient’s features, disease state, age, gender, lifestyle, genetic profile, etc. Beyond the design of implants, prostheses, implants porous scaffolds, reconstructed 3D-prototypes have been employed to investigate the pathophysiology of a disease. However, the medical field is taking a close interest in this technology with the possibility of creating solutions adapted to each patient [8]. There are approximately 7.5 billion diverse morphologies to which doctors must now adjust. AM then looks like a novel key for the creation of tailor-made devices that meet patients’ needs [9].

The American firm Allied Market Research estimates the market for 3D medical printing to be worth $2.3 billion by 2020. This growth would be clarified by the chances in terms of personalization offered by AM, whether to create prostheses, implants, to better prepare for a surgical operation or to fabricate medical products easing same complex operations such as surgical guides and other visual aids [10].

**Figure 4:**
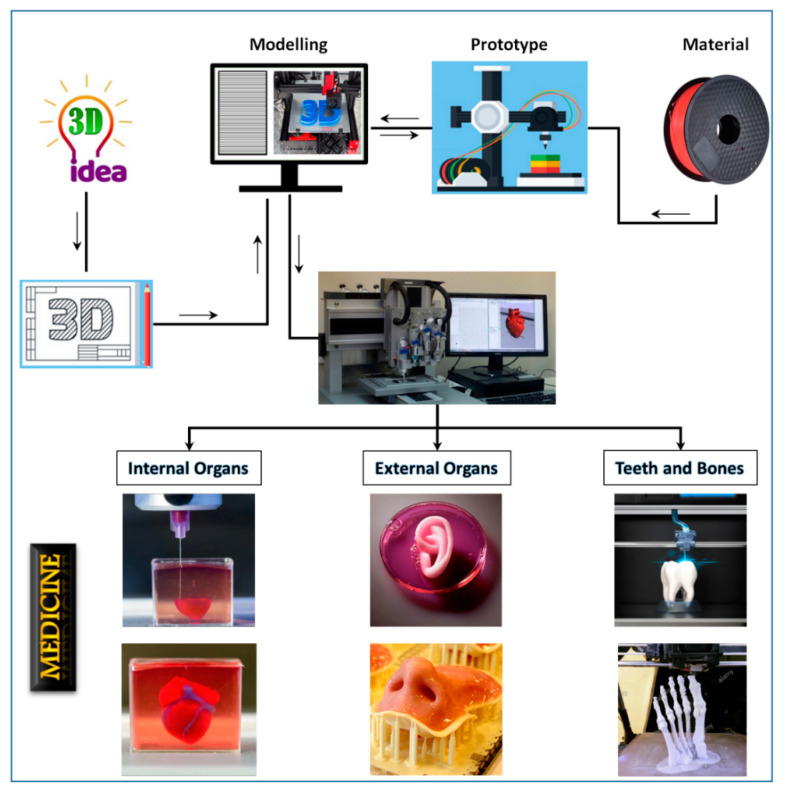
3D printing flowchart in medicine.

The purpose of AM for designing implants is to change an organ over a long period or to complement one or more of its utilities. Thus, by definition, it is entirely adapted to the patient and his or her anatomy. Customization is time-consuming and costly when by conventional manufacturing techniques. This is where AM makes sense and helps in the design of custom implants. Several players have entered this field and are using 3D technologies to manufacture customized medical products. The overall additive manufacture implant made from 2014 to 2026 is predicted to rise rapidly [11]. Figure 4 shows the different possibilities offered by AM for the field of medicine.

AM offers many opportunities for medical and health professionals, especially in the following areas:

1. The design of new products with the realization of functional prototypes: the bio-compatibility of certain materials enables patient testing and the fabrication of pathological organ prototypes to facilitate preoperative planning and analysis of surgical treatments.
2. The production of prostheses and implants: 3D printing offers the possibility to create even the most complex shapes of compatible polymer materials or titanium from scans or MRI scans. AM is ideal for the unit manufacture of complex parts.
3. Surgery: The 3D impressions produced are used as templates to prepare for surgery, identify areas of intervention, form any titanium parts, and finally prepare cutting or drilling templates. In addition to the time saved in the operating room (and the savings for the hospital), one can count on a faster recovery of the patient.
4. Medical research and education: 3D printing is also used in medical research to visualize and enable organ or concept manipulation.
5. Research on direct manufacturing of tissues with complete vital tasks [12,13]: although these applications are still far from widespread clinical implementation due to a number of fundamental method and scientific problems that remain to be solved, major scientific advancement and applications have been conducted in these areas.

Year after year, 3D printing provides ever more applications in the healthcare area serving to rescue and improve lives in ways never conceived up to now with COVID-19. In the pharmaceutical and medical industry, the focus is on improving product quality and reducing time to market for new products [14]. At all stages of product development, 3D printing improves and accelerates decision making upstream, the use of a 3D printer makes it possible to validate an idea, then a design and ergonomics based on iterations, and finally to test the product’s functionalities. Indeed, the robustness of the parts and their mechanical characteristics will make it possible to manipulate them or validate their handling. In surgery, the use of a 3D printer reduces the intervention time and improves the quality of the intervention and therefore the related costs (anaesthesia, operating room), and allows a better recovery by the patient [15]. Today, the main professionals using 3D printers in the medical world are:

- surgical departments and hospitals
- medical and pharmaceutical laboratories
- prosthetists
- universities
- teaching and education
- medical research

**Figure 5:**
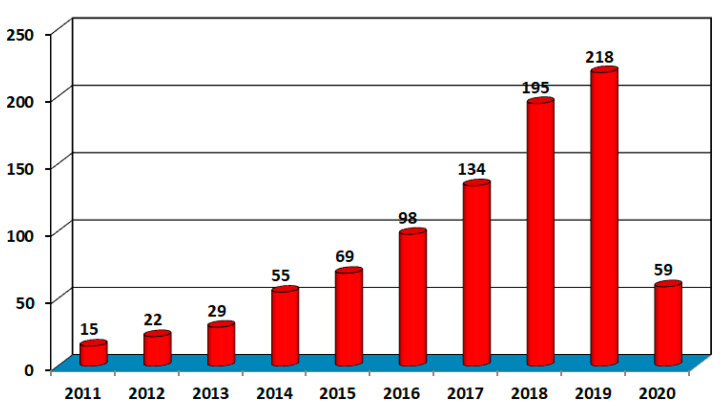
Publication of research papers in medical AM.

A scientific literature search is performed utilizing the Scopus database on the AM of medical applications. According to the Scopus database, the research in AM for medical application increased quickly from 2011 to April 2020. Total of 894 research papers published in this helpful area. In 2011, there were only 15 articles published in this field, and it rises to 894 by 2020. Figure 5.

Recently, many scientific researchers reviewed the application of AM technologies in medical and health care [16,17,18,19]. According to literature, several articles studied the AM application in the medical field and its advantages along with current and future applications. Table 1 presents the latest published articles in 2020 on the manufacture of medical devices using 3D printing.

The principal printing technologies employed in AM that can be differentiated depending on the nature of the material being treated are [24]: selective laser sintering (SLS), electron beam melting (EBM), selective metal sintering (SLM), stereolithography (SLA), direct metal tooling (DTM), fused deposition modelling (FDM), multijet/polyjet 3D printing, digital light processing (DLP), direct metal laser sintering (DMLS), selective deposition lamination (SDL), binding jetting/project 3D printing, and laminated object manufacturing (LOM). The large choice of AM techniques presently accessible, from FDM to SLM or stereolithography, provides excellent versatility in the fabrication of complex biomedical implants and parts. Depending on the final use, particular methods can be preferred. Table 2 shows the applications of the principal technology of AM in the medical field.

### 3.2. SWOT Analysis of AM in Medical Application

**Figure 6:**
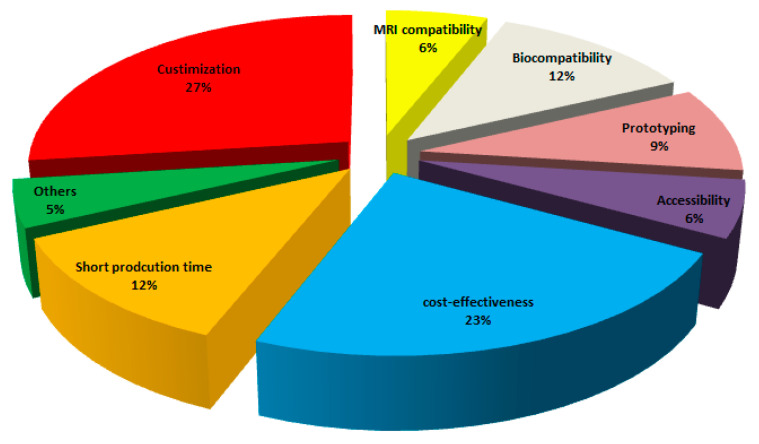
Reasons for the choice of AM in medical fields [27].

The AM technique is very consistent with the medical sector, since complicated bio-compatible parts can be synergistically manufactured with minimum restraints and a large degree of customization can be accomplished. Nevertheless, various considerations such as repeatability, efficiency, and smooth workflow need to be taken into account to acquire all the advantages of AM approaches. More sophisticated and customized components, that is, medical implants, can be made faster and cheaper with a sustainable AM pathway. AM use is expected to grow at an annual rate of approximately 16% by 2020 [27]. Figure 6 presents the reasons for the choice of AM in the medical fields.

There are also some main limitations of AM compared to conventional fabrication. The conventional manufacturing technique is generally much quicker for mass production. Products manufactured having restricted size depends upon machine print bed size. Per piece production cost can be high due to base material is costly and no economy of scale. Only skilled human resources are required to work these machines. Also, the materials involved for 3D printing of implantable devices are predominantly costly linked to those utilized in conventional fabrication techniques. Thus, the technique sets restrictions for the use of AM in fields where high material integrity and sophistication are required. After manufacturing of the part, there is a requirement of post-processing that increases the cost of the device. These problems must be solved with advances in materials science and engineering i.e., by expanding the selection of materials and therefore lowering the cost.

The AM in the medical area and design needs to think outside the norm for changing health care. The main pillars of this new technology are the capacity to deal more people where it previously was not possible and must be accompanied by an updated and current legislation to guarantee its correct use. Table 3 presents the SWOT analysis of AM in the medical field.

### 3.3. Implications of the COVID-19 on the Global AM Industry

Nowadays, many are wondering about the implications of the current COVID-19 pandemic on AM/3D printing as an industry. In response to that, the relationship between coronavirus and 3D printing technology is not entirely obvious. This is mostly because we are very far from understanding what the long, medium and short terms implications of this pandemic on global supply chains. However, one of the sectors that is anything but slowing down is the health and medical care sector. Medical staffs in hospitals are working under great pressure to treat the alarming number of COVID-19 patients that require medical assistance. An essential issue is that global stocks of medical equipment such as ventilators, masks or face shields are not adequate due to the high demand and general disruptions to the global supply chain, leaving many staff and patients without protection.

AM is likely to be able to play an important role in helping to support industrial supply chains that are influenced by shortages on conventional production and imports. One thing is most certainly though: 3D printing industry can have a straight away helpful effect when the supply chain is totally broken. In the following, one seeks to survey the 3D printing companies as well as academia around the world that embarked helping in the production of medical devices, accessories, components, and/or parts using 3D printing technology to fight the new COVID-19 pandemic. A brief review related to the latest 3D printing efforts against COVID-19 is illustrated in Table 4.

### 3.4. Research Status on Materials Used for the 3D-Printed Medical Parts

Questions arise in this regard, what AM materials can be used for medical parts? Should they be made of biocompatible materials that comply with health requirements such as those used in hospitals, and are they safe to use near the face in 3D printed masks for example? All that related to these issues will be discussed in the sequel.

First, one aims at providing a comprehensive overview on the materials mostly used in additively manufactured medical instruments than one review the materials used to print the medical supplies such as face masks for medical workers battling COVID-19. The basic material used in printing can be considered less expensive compared to the used printing technology which is frequently expensive. The choice of AM materials is directly related to the technology used. According to the literature, the major printing technologies used in AM of the medical instruments are: stereolithography (SLA), fused deposition modeling (FDM), material extrusion (ME), digital light process (DLP), powder bed fusion (PBF), material jetting (MJ), vat photopolymerization (VP) and binder jetting technology (BJ) [29,30]. The most widely used technology is material extrusion (ME) with 35% of the applications. Followed by PBF and MJ with 26% and 21%, respectively. Based on medical application, an essential factor to take into consideration is the biocompatibility of the medical device. The biocompatibility aspect is principally linked to the material used. In the literature, there are two principal polymers used, that are, polylactic acid (PLA) and a type of polyamide (PA2200), the use of which is rationalized by their biocompatibility. Due to the biocompatibility of the metals such as stainless steel, alumina-zirconia composites, or cobalt-chromium alloys, they are also employed to manufacture medical devices. Although it can be used for long term implantable devices, its use is limited compared to the polymer-based materials.

**Figure 7:**
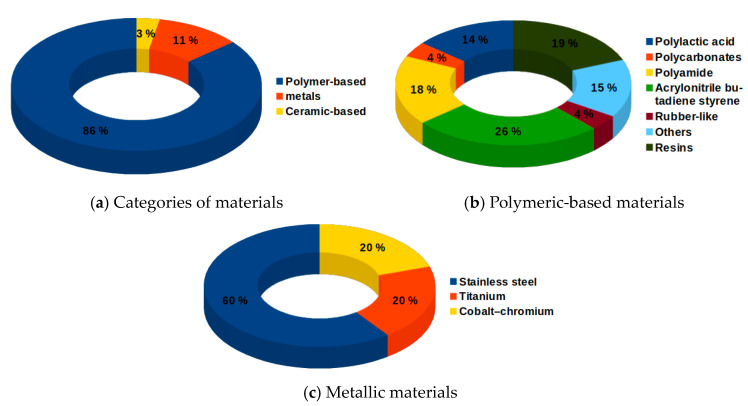
Classifications of materials used in printing medical devices.

The materials used in AM for medical devices can be broadly divided into three principal classifications: polymer-based, metals and ceramic-based. The category with the highest number of applications (86%) is that of polymer-based materials, Figure 7a. The Polymer-based materials comprise acrylonitrile butadiene styrene (ABS), polylactic acid (PLA), polyamides (nylon), polycarbonates (PC), resins, rubber-like materials, and polycaprolactone (PLC) which is classified as “others” [30,31,32,33,34,35,36,37,38,39], Figure 7b. The widespread use of polymer-based materials is mostly attributed to the biocompatibility, as for PLA, and biodegradability as in the case of polyamides raw powder PA2200 [34]. In comparison with polymer-based materials, metals are scarcely employed. Stainless steel (SS) is the most widely used, followed by both titanium (Ti) alloy and cobalt-chromium (Co–Cr) alloy [40,41,42], Figure 7c. Besides, it is found that the ceramic materials are used in different medical purposes: ceramic-filled epoxy resin and alumina-zirconia composite in the DragonFlex steerable laparoscopic grasping forceps instrument [43,44] and in the instruments dedicated to tracheostomy care and breathing-assisted surgical procedure [30]. Polonsky et al. [45] investigated the origins of complex grain morphologies and orientation gradients in additively manufactured stainless steel via the full 3D microstructural characterization. Zhao et al. [46] presented the effect of the thermal history during the PBF-EB process on the isothermal γ → ε phase transformation, aided by the numerical simulation of thermal history evaluation. Zhang et al. [47] studied the capacity of titanium–copper to override the negative effect of a high thermal gradient in the laser-melted region during additive manufacturing. In addition, AM can be used in metamaterial or microscale material design, for example [48,49].

Given the current state of emergency that is being encountered due to the spread of the COVID-19, impressive initiatives and solutions are emerging, especially to alleviate the shortage of medical instruments and personal protection equipment. Therefore, one aims here to present an overview of the materials as being through AM for use in a medical application in response to COVID-19 crisis and describe briefly the significant ones.

**Figure 8:**
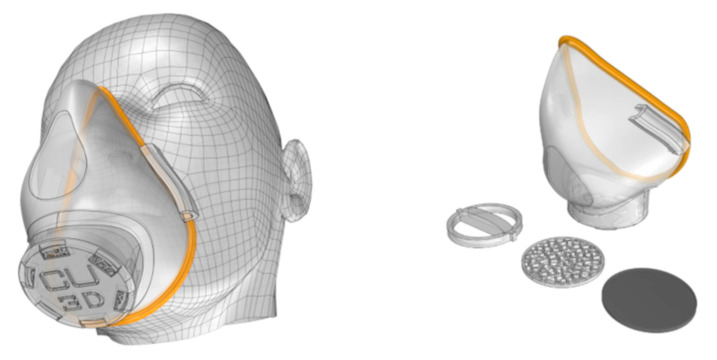
NanoHack mask printed with a recyclable and biocompatible polymer PLActive.

On 3D printing resource platforms and numerous pages of companies in the 3D printing sector, different mask models are shared for 3D printing. Recently, the Copper3D team has shared design of the mask, viz. NanoHack 2.0 [50], Figure 8. This mask is made up of a strong and hermetic mono-block structure, which is 3D printed with PLActive to provide maximum protection against the external environment. PLActive is a recyclable and biocompatible polymer that contains a copper nanocomposite that has shown antimicrobial properties. To make the mask watertight, the frame is sealed with a 3D printed edge with MDFlex, an antimicrobial TPU. MDFlex is an innovative nanocomposite developed with a high-quality TPU98A and a patented nano-copper additive, scientifically validated and highly effective.

**Figure 9:**
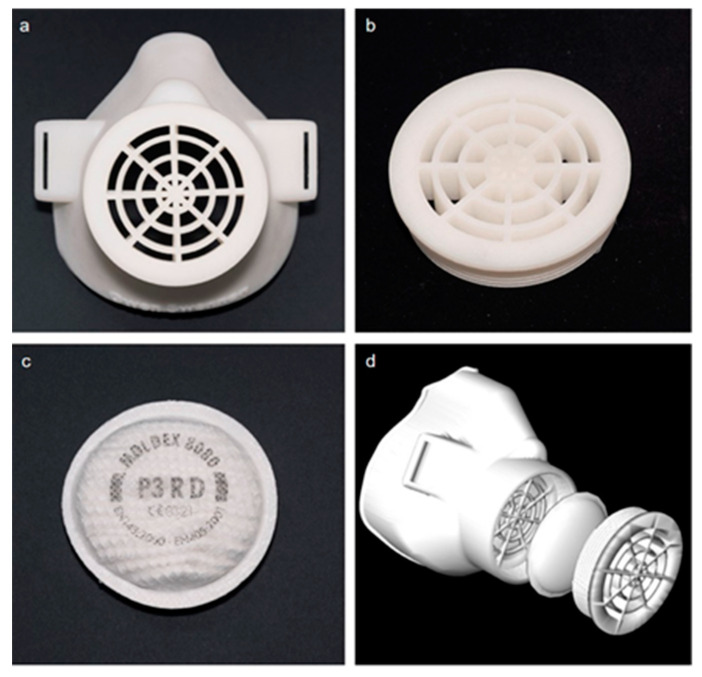
Custom made 3D protective face mask. (**a**) Reusable 3D-printed face mask, (**b**) filter membrane support, (**c**) filter membrane and (**d**) 3D image of the prototype.

In recent work, Swennen et al. [51] exposed proof of concept and prototype of a reusable additively manufactured protective face mask which can be adopted and used all over the world when needed. This mask is composed of two 3D-printed reusable components made of polyamide composite (face mask and filter membrane support) and other two disposable parts (head fixation band and filter membrane) as shown in Figure 9.

**Figure 10:**
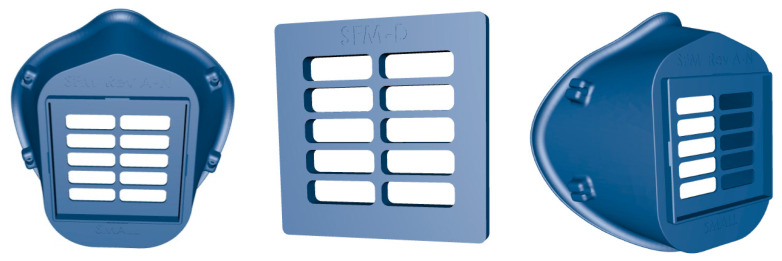
A stopgap face mask made of Nylon.

Created by the Veterans Health Administration (VHA) team, the 3D-printed surgical Stopgap face mask, is a personal protective mask for health care professionals that can be used for protection against liquid during COVID-19 emergency [52]. The clinicians have demonstrated the effectiveness of the 3D printed mask design for use in response to the shortage of medical supplies resulting from the coronavirus crisis. The mask is composed of the 3D printed mask and filter cover, two elastic strips, and a rectangular patch of filter material (see Figure 10). It is made of medical-grade nylon which is autoclavable and compatible with disinfectant cleaners and the 3D printing method is powder bed fusion via selective laser sintering. The printed mask is reusable and easy to disinfect, and it holds in place a disposable filter media. This mask has been approved by the Food and Drug Administration FDA to meet regulatory standards to protect clinical healthcare providers against COVID-19.

**Figure 11:**
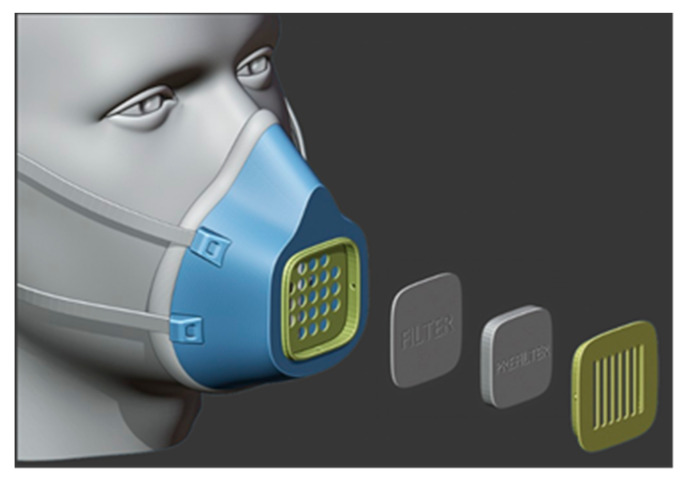
COVID-19 mask by Lafactoria3d made of PLA.

To temporarily alleviate the shortage of personal protective equipment, “Lowell Makes” has joined this movement and worked on developing 3D-printed masks, viz. COVID-19 mask [53], Figure 11. For printing this mask, it is recommended to use filaments (PLA, PETG) with low porosity and that offers the possibility of sterilization without damage (withstand high temperatures). PETG is a glycol modified version of polyethylene terephthalate (PET), which is a semi-rigid material with good impact resistance. PLA (Polylactic acid) is the most used material in 3D printing because of its easy printing and it has a low extrusion point (180–210 °C) and does not need a heated base. For correct operation of this mask, the pieces must be perfectly printed, without gaps between layers, and with the best merge possible between walls. This mask is not approved by any regulatory agency like FDA and has not passed any laboratory tests. Knowing that, Lowell Makes no guarantees that this mask will prevent transmission of COVID-19.

**Figure 12:**
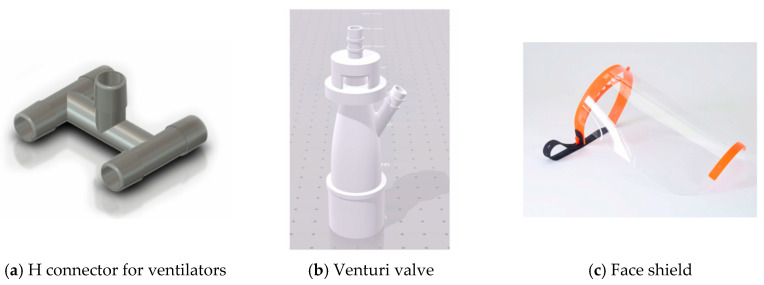
Valves and screens that can be printed using PLA, ABS, or PETG.

For the manufacturing of screens or valves that, due to their use, do not need to be composed of antibacterial or biocompatible materials, basic materials can be used in 3D printing, such as PLA or ABS, Figure 12. This is due to such elements that do not require specific properties, although it is evident that they require some rigidity and it’s not recommended to use flexible materials such as Flexfill.

### 3.5. Technical Considerations for the 3D-Printed Medical Devices

The 3D printing can be considered as a creative and flexible approach that can allow addressing access to vital medical products against COVID-19. For medical devices including personal protective equipment, 3D printing has the advantage of facilitating the fabrication of complex engineering structures that will not be easily feasible using non-AM approaches. While 3D printing can be used to print specific accessories and components, some complex components are not readily printed in 3D. As such, it might be helpful to use plans from original parts or those that have the same specifications and dimensions as they are accessible. Moreover, it is indispensable to affirm that any additively manufactured parts fit and work correctly as intended before using them in any clinical setting.

The PPE such as face shields, goggles, medical masks, face masks, and respirators is designed to protect the wearer’s body from injury or infection. Although 3D printing can be used to make certain protective equipment, few technical challenges should be overcome to be sufficiently effective. For instance, 3D-printed protective equipment can provide a physical barrier to the environment, but they are unlikely to provide the same level of barrier protection, fluid resistance, air filtration protection, and infection control as that of surgical masks and N95 respirators. For these reasons, healthcare providers are required to do the following when they are to use a 3D-printed mask: (i) examine the printed mask seal for leakages, (ii) ensure that the wearer of the mask can breathe through any makeshift filter materials, (iii) be careful in surgical settings where the need to protect against liquid and flammability is a concern, (iv) recognition that the mask may not have adequate air filtration to avoid transmission of infectious agents, and (v) safe disposal of infectious substances and disinfection of any part the mask wearer intends to reuse.

With the growing use of 3D printing and the uncertainty of how technology can influence the safety and performance of the products, interest in 3D printing has grown considerably. The purpose of this section is therefore to outline the main technical aspects and considerations related to 3D printing processes and recommendations for testing and characterizing the additively manufactured medical products. Within that scope, in October 2014, a public workshop was held to bring together a broad spectrum of stakeholders to discuss these technical aspects and considerations. This workshop was titled “AM of medical devices: An interactive discussion on the technical considerations of 3D Printing” [54]. At that meeting, discussions are separated into five broad technical themes: (a) materials; (b) design, printing, and post-printing validation; (c) parameters and characteristics of printing; (d) mechanical and physical evaluation of final products; and (e) biological aspects of the final devices, comprising biocompatibility, cleaning and sterilization. In 2017, FDA has released a new directive to share technical considerations of using 3D printing technologies in manufacture medical devices [55]. This guideline covered all stages ranging from the design stage to the test stage and sterilizing process.

**Figure 13:**
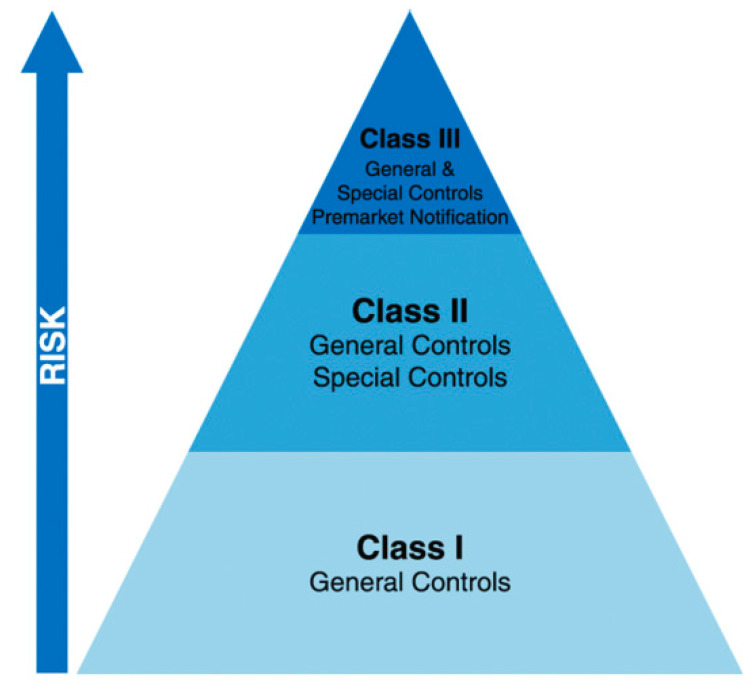
Categorization of medical equipment depending on the degree of risk to the patient.

In general, for a medical device to reach the general market, it must first pass a set of standards and regulations designated by its class. The devices are classified as Class I, II, or III based on the device’s risks and the level of control needed to ensure safety and efficacy, Figure 13. A large degree of risk requires greater regulatory control for approval of the device market. Thus, the first category devices (Class I) are deemed to be low-risk and normally subject to general controls alone, whereas the third category devices (Class III) that comprise most implants, are viewed high-risk and typically are subjected to the most complete and strict criteria [56]. Class III devices often aim to treat a scarce disease process.

In connection with these classes, the printed medical products used against COVID-19 may require far less material regulation, biocompatibility testing, and dimensional accuracy than AM produced biomedical devices and tissue-engineered scaffolds.

By FDA, most of the 3D printed devices have been reviewed as Class II devices. As stated earlier, the classification rules for devices are made according to the risks to health for a device’s intended use and the degree of control essential to furnish a reasonable assurance of the safety and effectiveness of the devices. Relying on the type of device, the use of 3D printing technique may introduce further technical concerns in terms of manufacturing controls, device performance, biocompatibility, and sterilization. Therefore, unless a 3D printed medical device shows a new or different question of safety or efficacy of the device type, the device will be categorized in the same regulatory category as other devices of that type, irrespective of the method of manufacture.

Preprinting considerations associated to 3D-printed medical devices, such as design control and device background, printing considerations such as raw materials and technical factors, and post-printing considerations such as cleaning, finishing and sterilization will be discussed subsequently.

**Figure 14:**
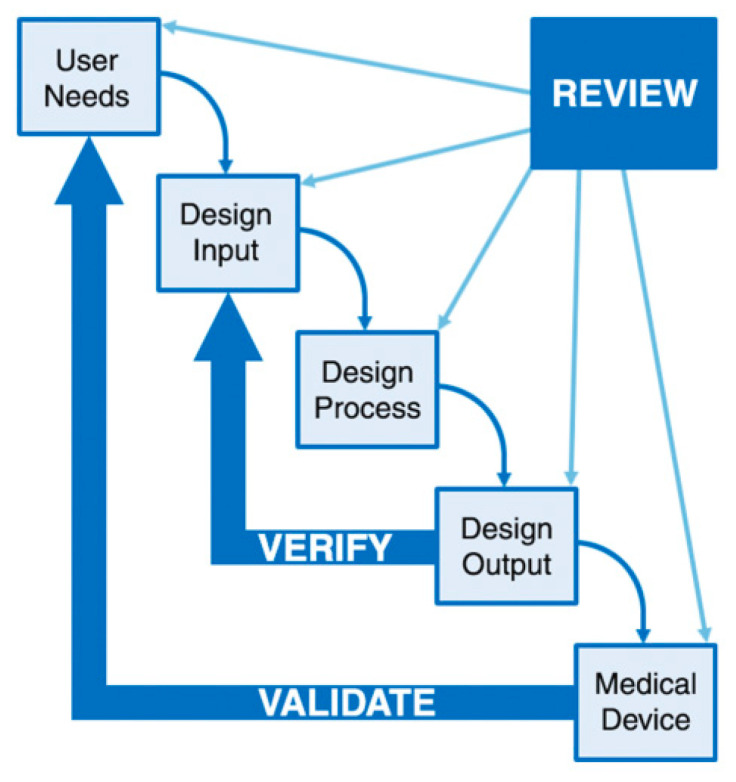
Design control model required for medical device development.

As a requirement to be considered for obtaining approval of the medical device is the design control. Design control designates the application of a formal methodology to the product development activities. It seeks to identify possible design flaws, create numerous design concepts, and verify and validate design effectiveness through repeated design review. In its basic form, the design control model is composed of the following correlated classes: user needs, design inputs, design processes, design outputs, design verification, design validation, and design reviews (see Figure 14). This design control model is suggested by the FDA for medical device development [55].

Concerning the raw materials, AM can use a range of different types of materials. It can be noticed that control of the material is an essential concern to guarantee successful manufacture, and that final performance of the device be significantly correlated with the material. Source materials for 3D printing should be evaluated, with appropriate quality control to ensure homogeneous and traceable manufacturing substrate. Regarding the technical considerations associated to 3D-printed medical products, a variety of 3D printing parameters have been found to have a major effect on the physical characteristics of the final fabricated product, such as scanning speed, laser beam energy density, deposition velocity, and humidity within the built environment.

The performance test shall be performed on final finished devices subjected to all post-processing, cleaning and sterilization processes. Concerning the post-printing considerations, cleaning of 3D-printed medical devices is needed irrespective of the 3D printing technique used. The cleaning method varies depending on the printing process utilized, from removing the support material to removing of residual monomers. The support material could be eliminated by physical or chemical methods. Some techniques might necessitate further finishing procedures such as sandblasting. The removal of remaining material through the cleaning procedure is possibly complicated in additively manufactured products that have porous regions or complicated internal geometries. Besides, such complex geometries are expected to increase the difficulty for cleaning and sterilization due to the likelihood of increased surface area, generation of extensive tortuous pathways. Also, it may be verified that the finishing operations do not change the global structure or mechanical characteristics of the fabricated product beyond the requirements of design input. Overall, material removal or cleaning process shall guarantee that remnant is eliminated to the level where it does not affect the safety or efficacy of the additively manufactured product.

Respecting the biocompatibility evaluation, the International Organization of Standards (ISO) has issued a group of guidelines for assessing the biocompatibility of materials employed in the fabrication of final finished medical products, conjointly called ISO 10993 [57]. These standards have been adopted by the FDA and they enforced them for Class III devices [58]. When trying to apply these criteria to additively manufactured implants, unique factors arise. The raw materials could be modified by the 3D printing process such as cross-linking, which would effectively modify the material properties. As such, testing of raw materials perhaps insufficient. Also, if chemical additives with known toxicities are used such as certain additives, catalysts, uncured monomers, and plasticizers, additional information may be necessary. However, concerning the biocompatibility evaluation in response to COVID-related respiratory and ventilator equipment, for instance, Stratasys Company performed a MED610 material assessment and found minimal biocompatibility risk in use for gas pathway components in respiratory and ventilator equipment. The assessment is carried out by experts in the field who have based their conclusions on a group of biocompatibility tests for which the material has been already certified and assessed the residual risk to satisfy ISO 18562-1 [59]. For coronavirus COVID-19 emergency scenarios, the risk assessment is, in particular when the material is used to print minority components in a dry or humidified gas path, where the product is not anticipated to be immersed in liquids.

The coronavirus disease (COVID-19) is a highly transmittable and pathogenic viral infection. This coronavirus is an RNA virus enveloped in a lipid bilayer [60]. Each virus particle has a small set of genes, enclosed by a sphere of fatty lipid molecules which looks like a spiky ball under the microscope. These viruses are the least resistant microorganisms on the scale of descending order of resistance to germicidal chemicals. Since sterilization operations kill or remove of all organisms and make products free from viable microorganisms, and because disinfection removes most pathogenic microorganisms, it is specifically possible to infer that sterilization and disinfection shall diminish the viability of the viruses on surfaces and in the air in enclosed areas.

Sterilization of additively manufactured products is essential to minimize the risk of infection from medical equipment. Because of numerous materials are used in AM, a diversity of sterilization processes can be used. It is possible to determine the sterilization process based on the device structure, availability of the process, and compatibility between the product material and process. The recommended COVID-19 sterilization and disinfection protocol for the cleaning and disinfection of 3D printed medical products including the facial shields indeed needs hygiene and virological verification and approval before use. A list of sterilizers and disinfectants for use against the coronavirus COVID-19 is reported in [60,61,62,63]. Sterilizers are medical devices for use in a health care facility controlled by FDA and are aimed to make medical products free from viable microorganisms. They differ based on the size designating from tiny sterilizers to massive sterilizers and method such as steam and evaporated hydrogen peroxide. On the other side, the disinfectant systems aim to use chemical or physical methods to destroy bacteria and certain forms of microorganisms. These devices can kill the majority of the recognized pathogenic microorganisms, but not essentially all microbial types like bacterial spores. In the framework of the COVID-19 public health crisis, it is essential to preserve sufficient stock of sterilizers and disinfectants that can promote the quick turnover of sterilized or disinfected medical instruments and that aid minimizing the risk of virus exposure to patients and health care workers for COVID-19.

In this regard, the Copper3D team has shared the recommendation for use of their recent 3D printed mask design NanoHack 2.0. The objective of this design is to provide protection to the population against particles in the air and to prevent the spread of liquid aerosols which can contaminate the respiratory tract. This mask is designed to be used in common spaces, for a maximum of eight hours, and to change the non-woven filter once a day. After handling the active filter, hands should be washed and the cleaning tips for NanoHack mask are specified as follows:

1. The device should be washed with soap and water.
2. Once washed, it should be rinsed well with clean water.
3. Equipment must be disinfected to inactivate any remaining pathogens. For this, chemical disinfection must be used, not using an autoclave, since PLActive (material from which the mask is printed) does not tolerate 80 °C or more.

In a recent work developed by Swennen et al. [51] to demonstrate a prototype of 3D printed face mask for healthcare against COVID-19, a cleaning and disinfection protocol is being suggested. As such, disinfection of the 3D printed mask is carried out using a solution with broad-spectrum antimicrobial action, with a 15 min exposure time (25 mL of Anios Clean Exel 0.5 in 5 L of water; DD Biolab S.L., Barcelona, Spain). This is performed in compliance with the official CORONA guidelines of the AZ Sint-Jan Hospital (Bruges, Belgium) with respect to cleaning and disinfection of eye-protective materials in COVID-19 units. The printed mask is rinsed with cold water after disinfection.

The most important consideration that can be inferred from this section is the answer to the frequently posed question about sterilization and disinfection of 3D printed parts in countering the spread of coronavirus disease (COVID-19). While it is possible to print complex devices such as face masks and valves we previously covered in previous sections, these devices must be sourced from original manufacturers whenever possible because they are made of materials that can be sterilized with heat and pressure in sterilizers. Because of the way these devices are manufactured, 3D printed materials are frequently more porous than typical medical device materials, allowing them to harbor microbes which means microbes can linger and grow into secondary problems if they are not carefully sterilized. It is important to not further harm people in attempting to help them. Indeed, medical sterilization techniques require heat, radiation, and chemical sterilization processes for bactericidal, virucidal and fungicidal effect. Therefore, any 3D printed device made for use with patients must be able to sustain repeated exposure to these processes. Most common 3D printing materials could easily warp, melt, or compromise the tensile strength and shape when exposed to medical sterilization. In this regard, it is disclosed that the most common sterile 3D Printing materials that can withstand the sterilization techniques are nylon PA, ABS and PEEK.

## 4. Conclusions and Final Remarks

The new coronavirus is an extremely contagious agent that causes fatal respiratory diseases, a major concern for global public health. There is currently no specific vaccine or treatment is available for patients diagnosed with COVID-19. In particular, there are growing efforts to prevent or reduce the transition to the frontline of health care providers. Nevertheless, there is increasing international concern with respect to the shortage in the supply chain of critical single-use PPE. With a severe lack of protective materials such as gloves, face masks, air-purifying respirators, goggles, face shields, respirators, and gowns. Additive manufacturing (AM) has emerged as a potential alternative solution. AM has the advantage of easing the manufacture of complex engineering structures such as medical devices including PPE that cannot be easily produced using conventional manufacturing methods. Given that AM is a possible savior to dwindling medical supplies in the fight against coronavirus, the present work has explored several issues about this technology that can play an important role in the medical device field. In this regard, an up-to-date review has been conducted to determine the capacity of AM for providing exclusive benefits to humanity within the medical healthcare supplies sector. Besides, the implications of the COVID-19 pandemic on the global AM industry were illustrated. The in-progress research of various materials that are used in AM for medical applications has also been explored and discussed. Despite the multiple benefits related to using AM in healthcare applications, there are some important limitations, and therefore the advantages and limitations of this technology have been presented. Furthermore, several inquiries related to technical aspects connected to AM processes, recommendations to test and characterize medical products that are additively manufactured, and biological issues related to final 3D printed products comprising biocompatibility, cleaning, and sterilization have been explored.

## Figures and Tables

**Table 1:** Latest publications in the medical field using additive manufacturing.

Authors	Description
T Cawley [20]	Use of 3D printing to manufacture a bone screw incorporating a porous surface to improve bony fixation
EH Backes [21]	Development of bioactive composites with biodegradable polymers using AM for medical applications
Eric Schwarzer [22]	Process development for AM of functionally graded alumina toughened zirconia components intended for medical implant application
Ashutosh Sharma [23]	Investigation of electrochemical corrosion behavior of additive manufactured Ti–6Al–4V alloy for medical implants in different electrolytes

**Table 2:** Applications of 3D printing methods in the medical field [24,25,26].

Method	Drug Formulation
**Fused Deposition Modeling (FDM)**	Tablets Oral dispersible films Capsules Customized medicine for drug delivery Dental fixtures, bridges and crowns
**Stereolithography (SLA)**	Tablets Personalised scaffolds Drug-loaded scaffolds Implantable devices Cell-containing hydrogels
**Selective Laser Sintering (SLS)**	Orodispersible tablets Oral drug delivery systems Accelerated release formulations Dental parts Medical parts Scaffolds

**Table 3:** AM in the medical fields—SWOT analysis.

**Strengths**	**Weaknesses**
Time reduction Sustainability Chain efficiency Better control of the construction process Minimizes transportation cost Part consolidation Light weighting parts Complex geometry Inner specifics	Expertise needed Production speed Less awareness Dimensional accuracy Post-processing needed Material limitation Cost of the AM technology printer Low production volume
**Opportunities**	**Threats**
Distributed Manufacturing Multi products batches Manufacturing on demand Potential growth market Design flexibility Allowing customization New product development	Less research collaboration with the industrial sector Reverse engineering Copyright problems/IP issues Regional and country regulations Dangerous weapons and security challenge

**Table 4:** 3D printing companies respond to COVID-19 [28].

3D Printing Companies	Number of Parts Produced
Consortium—Formlabs, Carbon, EnvisionTec, and Origin: nasophryngeal swabs (potential weekly capacity)	4,000,000
Nexa3D (3D printing manufacturer, United States): Test swabs (potential weekly production capacity)	500,000
Stratasys & Origin (United States): Nasopharyngeal swabs (Planned production capacity per day)	190,000
Nissan (car manufacturer, Japan): Face shields (potential weekly production capacity)	100,000
Voodoo Manufacturing (3D printing, United States): Face shields and swabs (weekly capacity for 2500 face shields and 50,000 swabs)	52,500
Ricoh 3D (Printing, UK): Face shields (weekly capacity)	40,000
3D Hubs (3D manufacturing, The Netherlands): Face shields (coordinated effort through the COVID-19 Manufacturing Fund)	20,000
Forecast 3D (Industrial 3D printing, United States): Face shields, stopgap masks, nasopharyngeal swabs, and other PPE products (daily part production capacity)	10,000
Nexa3D (3D printing manufacturer, United States): Face shields (potential weekly production capacity)	10,000
Prusa Research (3D printing company, Czech Republic): Face shields	10,000
Mobility/Medical goes Additive consortium (around 50 enterprises, Germany): Face shields	5000
PERA CD- N95 mask lining bracket—Farsoon Technologies (China) -Safety goggle & Mask adjuster. (Large- Safety googles- scale PPE manufacturer, China): (2000 daily)	-
Protolabs (3D printing company): Ventilator components	3000
Fast Radius (Additive manufacturing solutions, United States): Face shield kits (inital shipment, potential daily production capacity of 10,000)	1500
Azul3D (3D printing manufacturer): Face shields (Current daily capacity; Goal of 20,000 face shields per week)	1000
SmileDirectClub (Digital dentistry enterprise): Face shields (initial shipment; potential capacity of 7500 per day)	1000
Photocentric (3D printing company, UK): Valves for respirators (trial run; potential capacity of 40,000 per week)	-
Y Soft 3D (Enterprise solutions, Czech Republic): Face shields (daily production capacity)	500
Weerg & PressUP (Italy): Protective visors	500
BCN3D (3D printing manufacturer): Face shields (initial shipment with 2000 more planned to ship)	400
Formlabs (3D printing company, United States): Test swabs (300 in one batch; potential capacity of 75–150 k per day)	300
Photocentric (Photopolymer manufacturer, UK): Face shield parts (first batch of prints; potential daily capacity for 4860 parts)	200
Omni3D (Industrial 3D Printing, Poland): Face shields (daily capacity)	120
Consortium led by Leitat technology center (Zona Franca Consortium, Spain): Pieces for respirators (planned daily production)	
